# Inclusions properties at 1673 K and room temperature with Ce addition in SS400 steel

**DOI:** 10.1038/s41598-017-02478-6

**Published:** 2017-05-31

**Authors:** Fei Pan, Hao-Long Chen, Yen-Hsun Su, Yen-Hao Su, Weng-Sing Hwang

**Affiliations:** 1 Department of Materials Science and Engineering, National Cheng Kung University, Tainan, 70101 Taiwan; 2 Physics Department, Technische Universität München, Munich, 85748 Germany; 3 Physics Department, Ludwig-Maximilians-Universität München, Munich, 80799 Germany; 4 Department of Electronic Engineering, Kao Yuan University, Kaohsiung, 82151 Taiwan; 5 Steelmaking Process Development Section, China Steel Corporation, Kaohsiung, 81233 Taiwan

## Abstract

Inclusion species formed in SS400 steel with Ce-addition was predicted by thermodynamic calculation. The analysis of the inclusion morphology and size distribution was carried out by applying Scanning Electron Microscopy (SEM) and Transmission Electron Microscope (TEM). Nano-Fe_3_O_4_ particles were also found in cerium-deoxidized and -desulfurized steel and their shapes were nearly spherical. The complex Ce_2_O_3_ inclusions covering a layer of 218 nm composed by several MnS particles with similar diffraction pattern. Most importantly, the complex Ce_2_O_3_ characterized by using TEM diffraction is amorphous in the steel, indicating that Ce_2_O_3_ formed in the liquid iron and then MnS segregated cling to it.

## Introduction

Nonmetallic inclusions with high melting point in steel could be harmful in production of high grade wire, spring, and bearing steels^1, 2^. Inclusions larger than 10 µm are probable to lower the yield strength obviously and also decrease tensile strength^3^. However, the yield strength and tensile strength would increase remarkably for steels with inclusions less than 0.3 µm^3^. Adding a or several metal elements into steel has been exploited by steel manufactures in order to form finer grain structures in steel and to improve the performance of the steel^4^. Titanium^5^, Aluminium^6^ and Magnesium^7^ have been studied a lot as deoxidizers and their functions have also been researched a lot to improve the strength of steel. Titanium influences on the intra-granular nucleation of ferrite only when the amount of sulfur in low carbon steels is extremely low^5^. The TiN particles found in Ti-killed low carbon steel are the most effective inclusions to promote intra-granular ferrite nucleation due to the crystal coherency of TiN with ferrite^8^. The change of Mg amount in low carbon steel would affect the mean size of inclusions, decreasing from 2.1 μm to 1.2 μm due to the wettability of oxides containing magnesium^9^. What’s more, the microstructure of Mg-deoxidized steel would be improved because of the heterogeneous nucleation of acicular ferrite^9^. Despite of the strong deoxidization ability of aluminium, the deoxidization of aluminium has a limit (a[O] 140–280 ppm) and the formed large inclusions (Al_2_O_3_) would cluster^10^. However, as reported, Ti, Mg and Al cannot desulfurize steel to a very low level^5–7^ and their ability of deoxidization is limited. Moreover, the techniques to add magnesium in liquid steel are not mature to obtain stable Mg-added steel.

Rare earths have attracted a lot of attention recently^11–16^. Cerium, a typical rare earth element, has been used as an important deoxidizer in many steels to improve mechanical properties by grain refinement^2^. Cerium is very reactive with oxygen and sulphur to form various species of inclusions, hence cause nozzle clogging and defects on steel surface^17, 18^. Due to the atomic properties of cerium, it has strong affinity to sulphur or oxygen and will, almost always, form sulfides or oxides when both of them are present in liquid steel^19, 20^. CeS particles with a fine distribution in steel would shift the process of nucleation from austenite grain boundaries to intra-granular sites when transforming from austenite to ferrite^21^. But this process has been studies extensively in low alloy steel weld metals studying the contribution of sulfide and oxide inclusion to acicular ferrite formation^22, 23^. Ce_2_O_3_, a typical inclusion formed in cerium-deoxidized steel, has the possibility to act as nucleation site for IAF during fast cooling, due to its low misfit value with ferrite^24^. Nevertheless, the characteristics and its microstructures of the inclusions formed in cerium-deoxidized and –desulfurized at high temperature and after furnace-cooling have not been studied yet.

This paper focuses on (1) verifying the thermodynamic calculations for main inclusions formed at high temperature and room temperature in SS400 steel; (2) the morphologies of inclusions formed during furnace-cooling; (3) the size distribution of inclusions formed during furnace-cooling. The mechanism for inclusions size distribution is also analyzed when the added cerium varies in liquid iron. What’s more, a model for inclusion sizes distribution is established for SS400 steel with cerium addition.

## Experiments

The experimental procedure sketch is illustrated graphically in Supplementary Figure S1.

### Raw steel preparation

Raw SS400 steel was prepared by using a high frequency induced vacuum furnace at National Chung Hsing University. Before heating, the furnace had to be evacuated to 7 × 10^−2^ torr, and then argon was used to keep the inner pressure of the furnace at 600 torr. The sample was kept in the furnace for 15 minutes after the iron powder was melted, and then the power was turned off. The steel was quenched when it was cooled down to 500 °C. The raw steel was cut into small pieces for secondary melting.

### Secondary melting experiment

The secondary melting experiment was conducted by using a high frequency induced melting furnace. The cut raw steel had to be polished to remove the oxides on its surface and then washed with alcohol, followed by weighting. A hole was drilled at the top of the washed raw steel, and cerium powder was poured in. Then, the steel bulk was settled in an alumina crucible, and they were placed together into a graphite crucible settled in a high frequency induced melting furnace. Argon was used to protect the inner atmosphere of the furnace from oxygen. The inner temperature of the furnace was held for five minutes when it reached 1073 K, and then the power of the finance was turned off five minutes later when the steel melted completely. The heating pathway is as shown in Supplementary Figure S2.

## Results and Discussion

### Verification of thermodynamic calculation by experiments during furnace cooling and quenching

**Figure 1:**
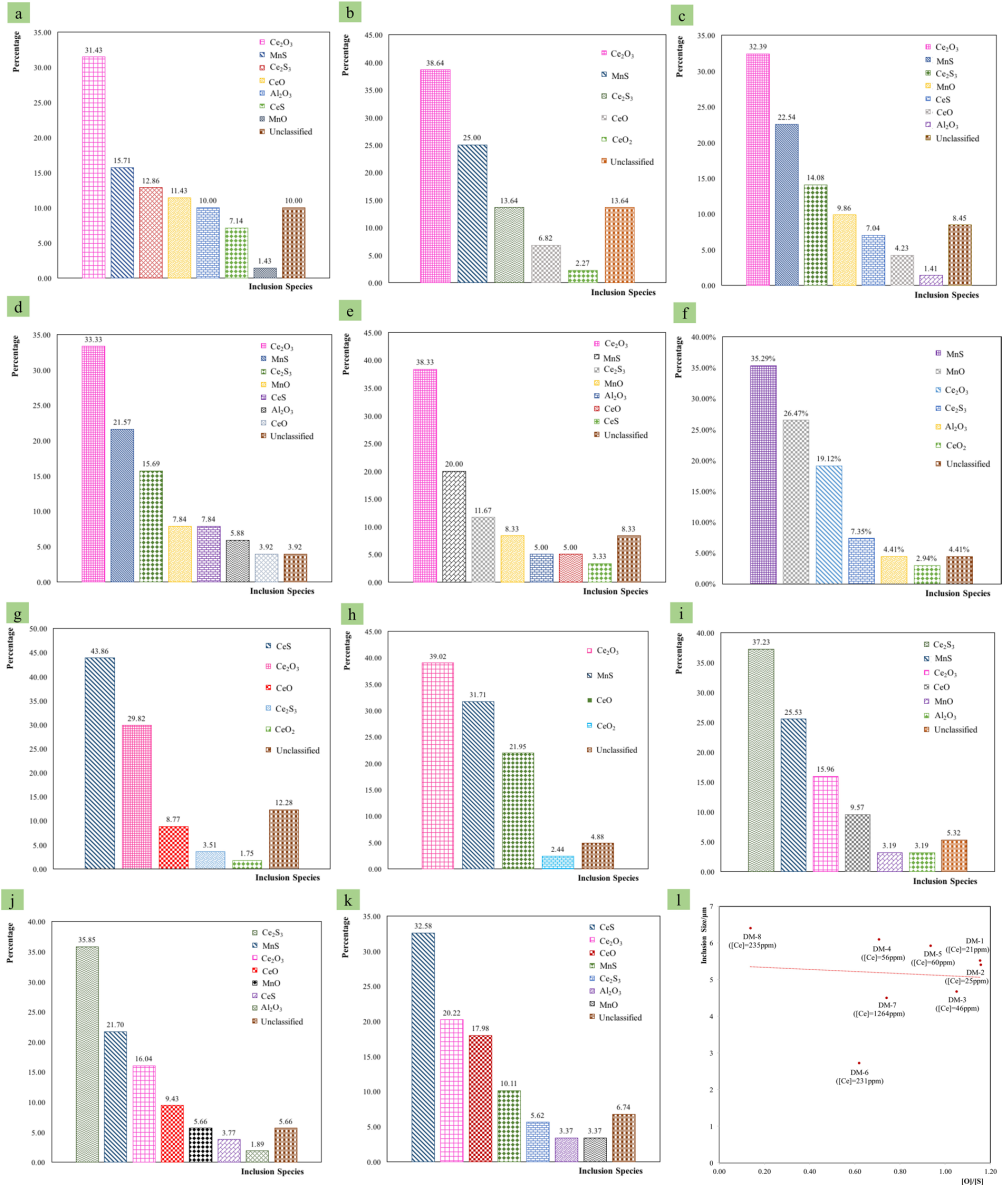
(**a**–**h**) Inclusion species distribution of different samples during furnace-cooling to room temperature; (**i**–**k**) inclusion species distribution for sample DM-2, DM-5 and DM-7 after quenching at 1673 K; (l) inclusion size distribution affected by oxygen to sulfur ratios of different samples during furnace-cooling to room temperature.

The verification of the thermodynamic calculation by applying equilibrium module of FactSage was realized by preparing several different SS400 samples with different chemical composition as shown in Table 1. And the samples were produced by adding different amount of cerium powder in 400 g liquid SS400 steel. The thermodynamic calculation results were obtained as shown in Table 2 by inputting the chemical composition of the steel samples. The chemical composition of different samples was detected by N/O analyser, ARL 4460 (optical emission spectrometer) and ICP-AES (inductively coupled plasma optical emission spectrometry). 21 ppm cerium were detected for sample DM-1, which was added 0.4 g cerium powder in the raw steel. Because of the limitations of the experiment, the inclusion formed at 1873 K could not be obtained by our experimental equipment. Therefore, the inclusions, which are used to verify the thermodynamic calculation of solidified steel, were obtained after solidification by furnace cooling. Based on the calculation of the solidification temperature, it was obviously found that the liquid steel would completely solidify below 1770 K. It is therefore reasonable to verify the thermodynamic calculation by using the obtained inclusions, which were formed after complete solidification. For the DM-1 sample, Ce_2_O_3_, MnS and Ce_2_S_3_ were the main inclusions after solidification by thermodynamic calculation (see Table 2). The SEM-EDS results (see Fig. 1(a)) of the DM-1 samples show the consistency between the experiments and the thermodynamic calculation. The results of thermodynamic calculations of samples DM-2 to DM-8 also show the consistency with the corresponding experiments.

**Table 1:** SS400 steel samples with different chemical compositions.

Experiment Sequence	[C]/%	[Si]/%	[Mn]/%	[P]/ppm	[S]/ppm	[O]/ppm	[Ce]/ppm	[O]/[S]	Added Cerium/g	Yield Ratio/%
DM-1	0.159	0.196	0.503	106.4	62.4	72	21	1.154	0.4	2.1
DM-2	0.199	0.197	0.505	112.7	12.1	14	25	1.157	1	1
DM-3	0.197	0.2	0.506	111.3	45.7	48	46	1.05	0.3	6.2
DM-4	0.195	0.204	0.509	117.1	75	53	56	0.935	0.1	22.6
DM-5	0.197	0.201	0.498	107.4	24.6	23	60	0.707	0.8	3
DM-6	0.013	0.201	0.827	13.4	147.3	91	231	0.618	1.5	6.2
DM-7	0.005	0.201	0.834	!1.2	31.5	23.3	1264	0.74	2.5	18
DM-8	0.101	0.397	1.366	64	49	6.7	235	0.137	4	2.4

**Table 2:** Results of thermodynamic calculations for the main inclusions formed in SS400 steel during furnace-cooling from 1873 K to room temperature and Quenching from 1673 K.

Temperature	Sample	Main inclusion species
Furnace-cooling from 1873 K to room temperature	DM-1	Ce_2_O_3_, MnS
	DM-2	Ce_2_O_3_, MnS
	DM-3	Ce_2_O_3_, MnS
	DM-4	Ce_2_O_3_, MnS
	DM-5	Ce_2_O_3_, MnS
	DM-6	Ce_2_O_3_, MnS, MnO
	DM-7	Ce_2_O_3_, MnS, CeO
	DM-8	Ce_2_O_3_, CeS
Quenching from 1673 K	DM-2	Ce_2_S_3_, MnS
	DM-5	Ce_2_S_3_, MnS
	DM-7	Ce_2_O_3_, CeS, CeO

DM-2, DM-5 and DM-7 were selected to verify thermodynamic calculation at 1673 K. At this point, a Confocal Laser Scanning Microscope (CLSM) was used to prepare samples quenched from 1673 K. After quenching from 1673 K, SEM was used to analyse the inclusions formed at 1673 K. The main inclusions formed at 1673 K are shown in Fig. 1(i–k). The main inclusions of DM-2 formed at 1673 K were Ce_2_S_3_, MnS, Ce_2_O_3_ and CeO, which is closely related to thermodynamic calculation at 1673 K, as shown in Table 2. The main inclusions formed at 1673 K of DM-5 were Ce_2_S_3_, MnS, Ce_2_O_3_ and CeO in Fig. 1(j), and the main inclusions formed at 1673 K of DM-7 were CeS, Ce_2_O_3_, CeO and MnS in Fig. 1(k). The main inclusions formed at 1673 K of DM-5 and DM-7 strongly confirmed the thermodynamic calculation of DM-5 and DM-7 at 1673 K, as shown in Table 2.

All these findings indicate that the thermodynamic calculation by using equilibrium module of FactSage is an applicable method to simulate inclusion formation during steel solidification especially in the case of steel with cerium additions. What’s more, the main inclusions formed in the steel after solidification were Ce_2_O_3_, MnS and Ce_2_S_3_ when the successful addition of cerium in steel was below 60 ppm, and Ce_2_O_3_ always formed during solidification when the cerium addition was below 1264 ppm.

### Effects on inclusion species and size by the added cerium and [O]/[S]

**Figure 2:**
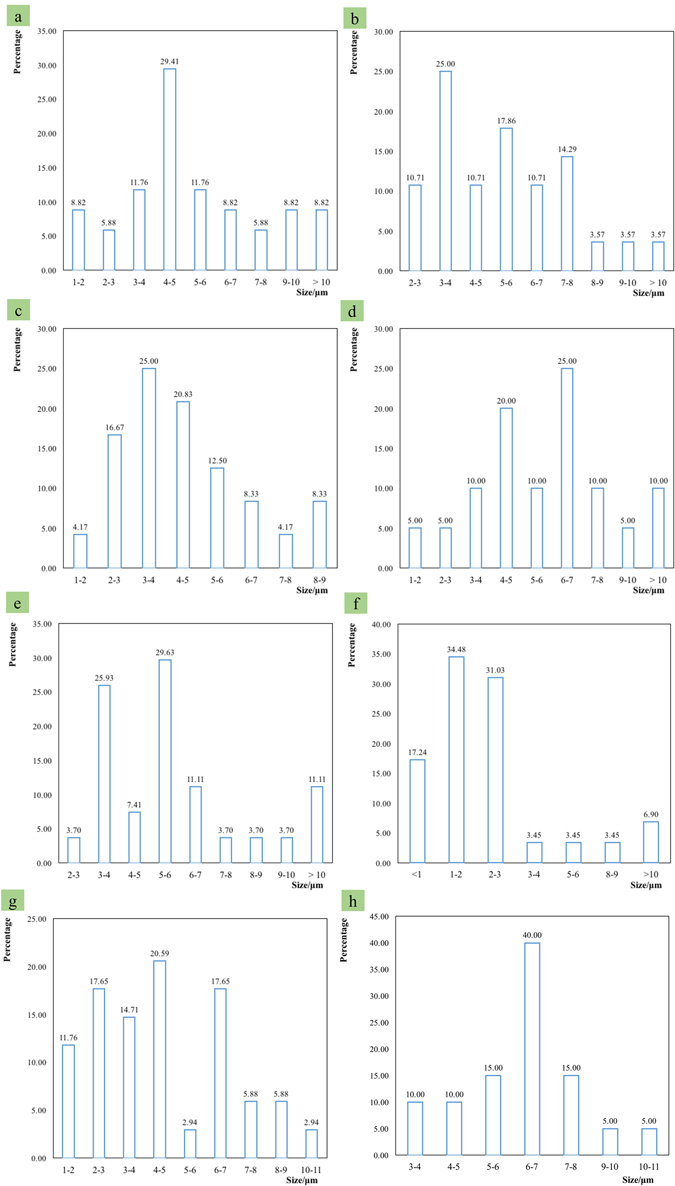
(**a**–**h**) Inclusion size distributions for different samples during furnace-cooling to room temperature.

The inclusion size distribution of different samples is shown in Fig. 2. For sample DM-6 with [O]/[S] of 0.618, the main inclusion size ranges are 1–2 μm and 2–3 μm, and the average inclusion size is 2.73 μm. For sample DM-7 with [O]/[S] of 0.74, the main inclusion size ranges are 2–3 μm, 4–5 μm and 6–7 μm, and the average inclusion size is 4.51 μm. For sample DM-8 with [O]/[S] of 0.137, the main inclusion size ranges are 6–7 μm, and the average inclusion size is 6.41 μm. For sample DM-2 with [O]/[S] of 1.157, the main inclusion size ranges are 3–4 μm and 5–6 μm, and the average inclusion size is 5.41 μm. For sample DM-1 with [O]/[S] of 1.154, the main inclusion size ranges are 4–5 μm, and the average inclusion size is 5.53 μm. For sample DM-3 with [O]/[S] of 1.05, the main inclusion size ranges are 3–4 μm, and 4–5 μm, and the average inclusion size is 4.68 μm. For sample DM-4 with [O]/[S] of 0.935, the main inclusion size ranges are 4–5 μm and 6–7 μm, and the average inclusion size is 6.11 μm. For sample DM-5, with [O]/[S] of 0.707, the main inclusion size ranges are 3–4 μm and 5–6 μm, and the average inclusion size is 5.93 μm. As shown in Fig. 1(l), it can be derived that the inclusion size tends to be 5 μm with the increasing [O]/[S].

**Figure 3:**
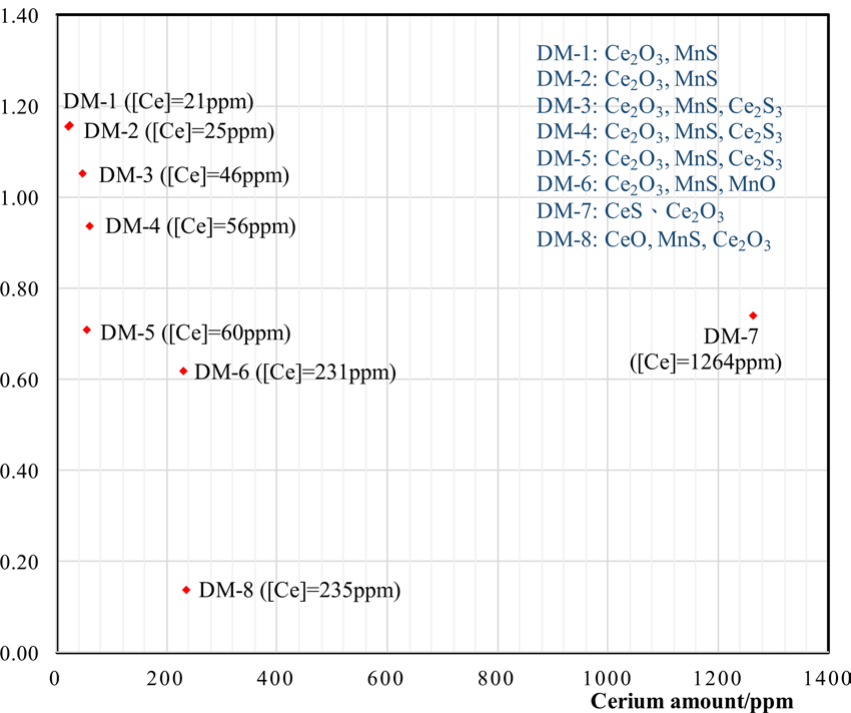
Inclusion species affected by amounts of cerium and oxygen to sulfur ratios in different samples.

The Fig. 3 indicates that DM-2 and DM-1 samples have very similar inclusion species based on their similar cerium amounts and [O]/[S]. Another important fact concluded from Fig. 1(l) is that the similarities in the inclusion species are very close in DM-3, DM-4 and DM-5, for which it is likely that the cerium amount and the relatively narrow [O]/[S] from 0.7 to 1.05 would be the main reason. The fact that DM-5, DM-6 and DM-7 have similar [O]/[S] shows they all have Ce_2_O_3_, but there is a divergence for DM-7 over the other two samples, which is the CeS rather than MnS existing in DM-7. The reason for this is that the amount of Cerium in DM-7 is much larger than that in DM-6 and DM-5.

### Morphological and compositional analysis of Ce-inclusion

The morphologies of the different inclusion species from the various samples are shown in Figure S3 to Figure S11. The morphologies for Ce_2_O_3_ appearing in samples from DM-6 to DM-5 are almost bright projections with random shapes. There are two types of MnS inclusions. The first kind of MnS is an outer covering of Ce_2_O_3_ (refer to Figure S8(a), Figure S9(d), Figure S10(d)) or Ce_2_S_3_, as shown in Figure S4(d), Figure S5(d), Figure S6(c) and (d) and Figure S7(c) and (d). Another morphology of MnS looks like a sphere as shown in Figure S3(b), Figure S4(b), Figure S8(b) and Figure S10(b). The observed CeS in Figure S9(a) embedded in the steel substrate appears to be different from the Ce_2_O_3_. The inclusion CeO appears like an embedded sphere in Figure S3(d) and Figure S9(b), but in Figure S10(c), it looks detached from the steel substrate. The morphologies of Ce_2_S_3_ can be classified into two categories: one is embedded gray particles, as shown in Figure S4(c) and (d), Figure S3(c), and Figure S7(c) and (d), and the other is bright, flat particles as shown in Figure S5(c) and (d) and Figure S6(c) and (d).

**Figure 4:**
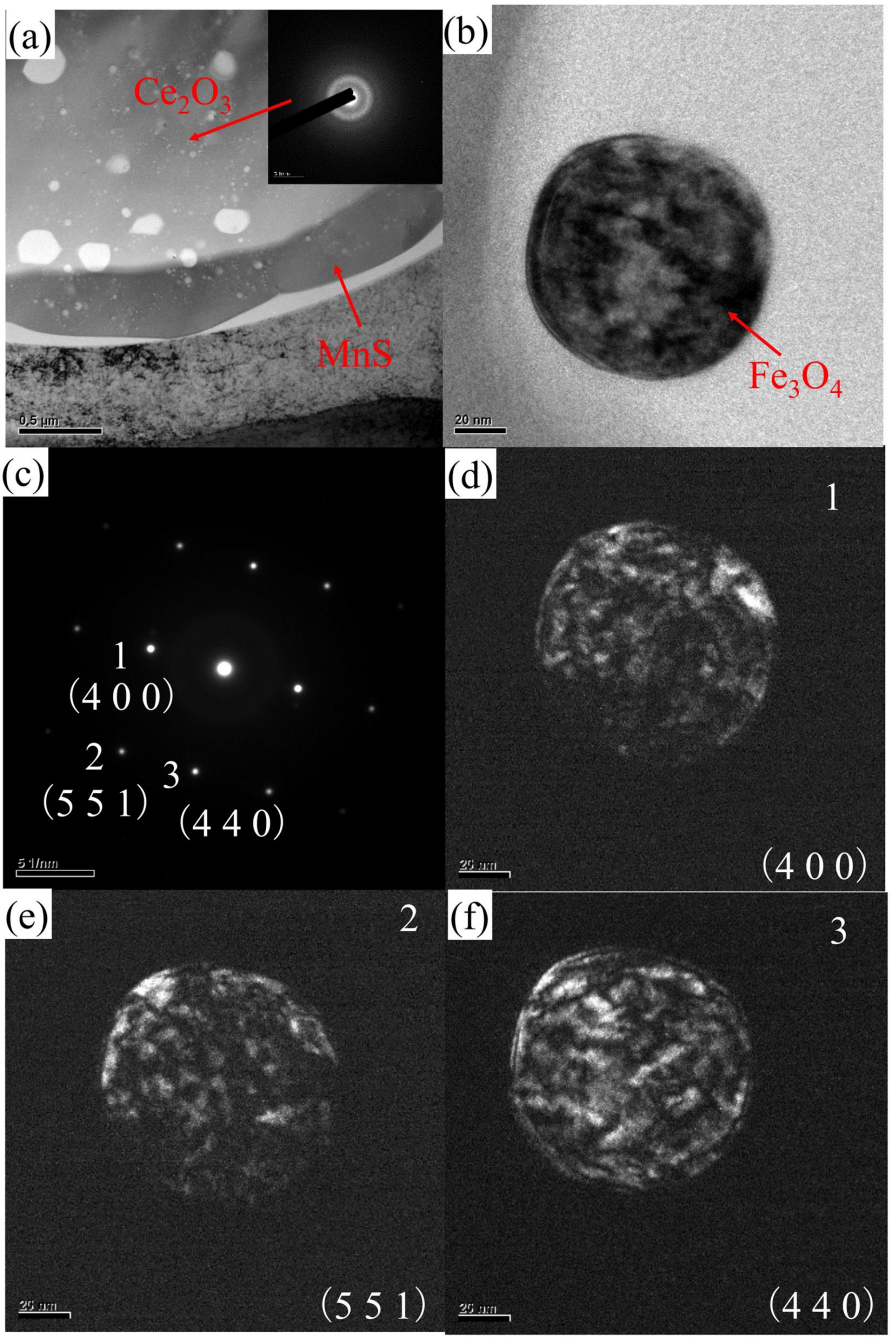
(**a**) TEM results of the complex inclusion found in sample DM-6, (**b**) morphology of nano Fe_3_O_4_ found in sample DM-6 and (**c**–**f**) SAD results of nano Fe_3_O_4_ found in sample DM-6.

Figure S8(a) indicates the morphology of Ce_2_O_3_ is not as easy as seen. The TEM analysis, which confirms the above, conveys a lot of valuable information about inclusion morphology. The TEM samples were prepared by FIB, as illustrated in Figure S11. Two inclusions like Figure S8(a) were found using FIB and prepared for TEM analysis (see Figure S11). Because of the diffraction pattern shown in Fig. 4(a), the Ce_2_O_3_ in the complex inclusion can be considered as amorphous structure. The amorphous Ce_2_O_3_ in the steel indicates that Ce_2_O_3_ formed in the liquid iron and then MnS segregated cling to it. What’s more, the nano-Fe_3_O_4_ particles were found ranging from several nanometers to about 180 nm. According to the SAD analysis with TEM, the shapes of the nano-Fe_3_O_4_ particles are nearly spherical, as shown in Fig. 4(d–f). Most importantly, the Ce_2_O_3_ covering layer is a thin MnS layer of about 218 nm. The MnS particles around the Ce_2_O_3_ have the same diffraction pattern, which indicates they have the same crystal structure.

## Conclusion

In this research, a thermodynamic calculation method was applied to predict the formation of inclusions influenced by the amount of cerium, which was added into liquid steel. Scanning Electron Microscopy (SEM) and Transmission Electron Microscope (TEM) were used to analyse the morphologies of the main inclusions formed in the produced steel. Chemical compositions of the inclusions and prepared steel were analysed by SEM-EDX, and the morphology of inclusions were characterized. The following can be concluded:

Thermodynamic calculation is an effective tool to predict inclusion formation during the solidification of liquid iron with the addition of cerium. The inclusion size trends to be 5 μm with the increase of [O]/[S] from 0.137 to 1.157. When [O]/[S] is around 1.15 and [Ce] amount is from 21 ppm to 25 ppm, the main inclusion species formed in SS400 steel are Ce_2_O_3_ and MnS. The main inclusion species would be Ce_2_O_3_, MnS and Ce_2_S_3_, when the cerium amount ranges from 46 ppm to 60 ppm with a relatively narrow [O]/[S] from 0.707 to 1.05. While the [O]/[S] in steel ranges from 0.618 to 0.74 and [Ce] is from 60 ppm to 1246 ppm in steel, it shows Ce_2_O_3_ could always form but MnS would transform to CeS with the increasing [Ce] in SS400 steel. Nano-Fe_3_O_4_ particles were found ranging from several nanometers to about 180 nm. According to the SAD analysis with TEM, the shapes of the nano-Fe_3_O_4_ particles were nearly spherical. Most importantly, the Ce_2_O_3_ covering layer is a thin MnS layer of about 218 nm. The MnS particles around Ce_2_O_3_ have the same diffraction pattern, which indicates they have the same crystal structure. Moreover, the amorphous Ce_2_O_3_ found in the steel indicates that Ce_2_O_3_ formed in the liquid iron and then MnS segregated cling to it.

## Electronic supplementary material

Inclusions properties at 1673 K and room temperature with Ce addition in SS400 steel
