# Supplementary material for: Inclusions properties at 1673 K and room temperature with Ce addition in SS400 steel
Source: Sci Rep. 2017 May 31;7:2564. doi: 10.1038/s41598-017-02478-6 (PMC5451397; doi:10.1038/s41598-017-02478-6)
Supplement: Supplementary file 1 — Inclusions properties at 1673 K and room temperature with Ce addition in SS400 steel [file 41598_2017_2478_MOESM1_ESM.doc]

**Supplementary Information**

**Inclusions properties at 1673 K and room temperature with Ce addition in SS400 steel**

Fei Pan^1, 2, 3, *^, Hao-Long Chen^4^, Yen-Hsun Su^1^, Yen-Hao Su^5^, Weng-Sing Hwang^1, *^

1. Department of Materials Science and Engineering, National Cheng Kung University, Tainan 70101, Taiwan;
2. Physics Department, Technische Universität München, Munich 85748, Germany;
3. Physics Department, Ludwig-Maximilians-Universität München, Munich 80799, Germany;
4. Department of Electronic Engineering, Kao Yuan University, Kaohsiung 82151, Taiwan
5. Steelmaking Process Development Section, China Steel Corporation, Kaohsiung 81233, Taiwan;

Email: [phoenix.pan@tum.de](mailto:phoenix.pan@tum.de) (F.P.); [t11033@cc.kyu.edu.tw](mailto:t11033@cc.kyu.edu.tw) (H.-L.C.); [yhsu@mail.ncku.edu.tw](mailto:yhsu@mail.ncku.edu.tw) (Y.-H.S.); [150151@mail.csc.com.tw](mailto:150151@mail.csc.com.tw) (Y.-H.S.); [wshwang@mail.ncku.edu.tw](mailto:wshwang@mail.ncku.edu.tw) (W.-S.H.)

***** Corresponding authors: [phoenix.pan@tum.de](mailto:phoenix.pan@tum.de) (F.P.) and [wshwang@mail.ncku.edu.tw](mailto:wshwang@mail.ncku.edu.tw) (W.-S.H.); Tel.: +886-6-2757575 (ext. 62928) (F.P. and W.-S.H.); Fax: +886-6-2344393 (F.P. and W.-S.H.)


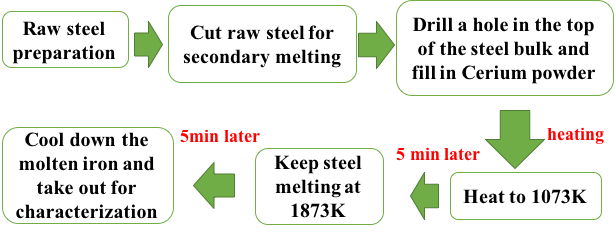


Supplementary Figure S1 Experimental procedures


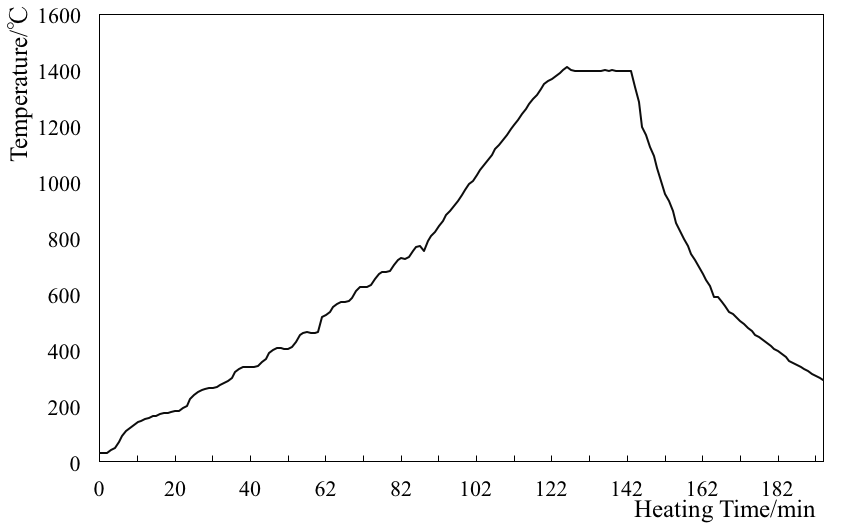


Supplementary Figure S2 The heating pathway for the secondary melting experiment


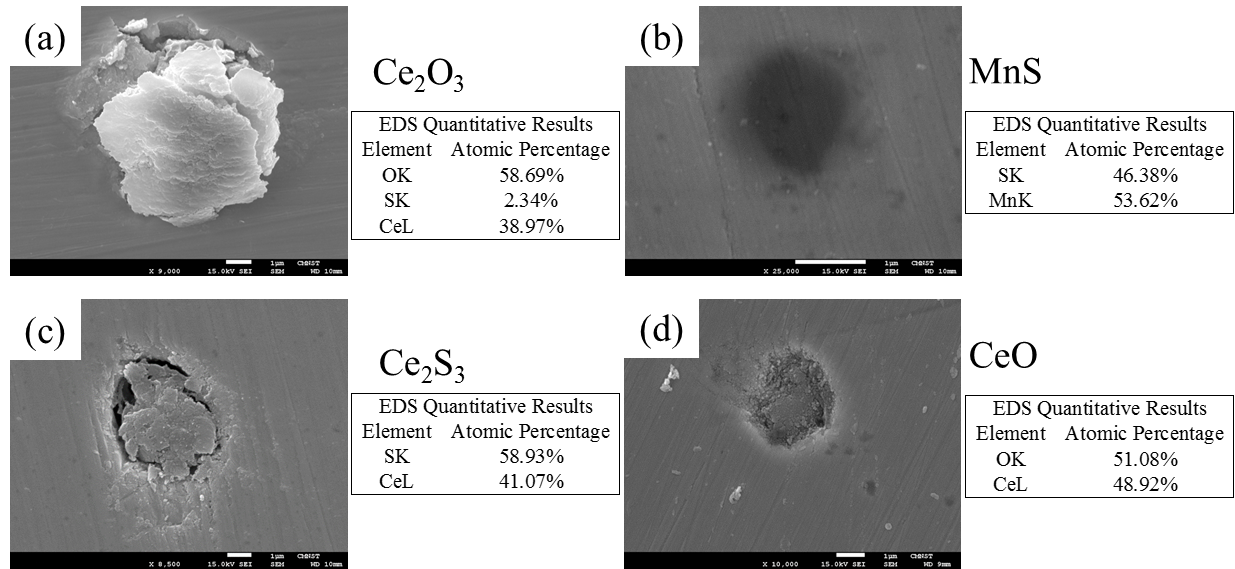


Supplementary Figure S3 Inclusion Morphology for sample DM-1


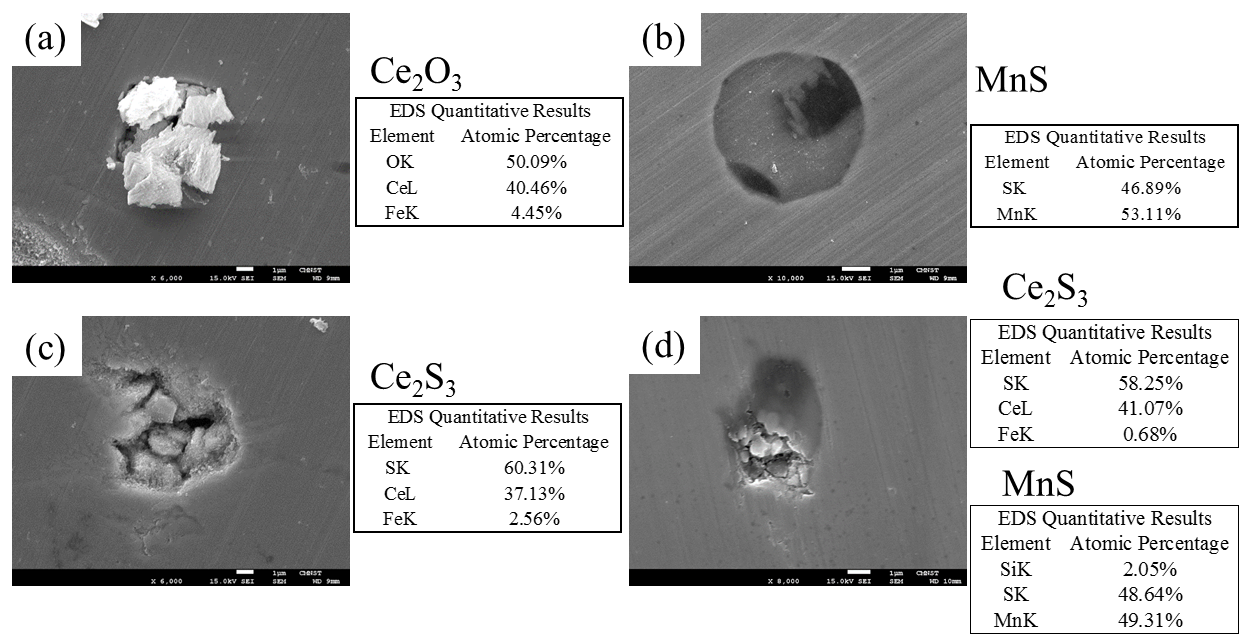


Supplementary Figure S4 Inclusion morphology for sample DM-2


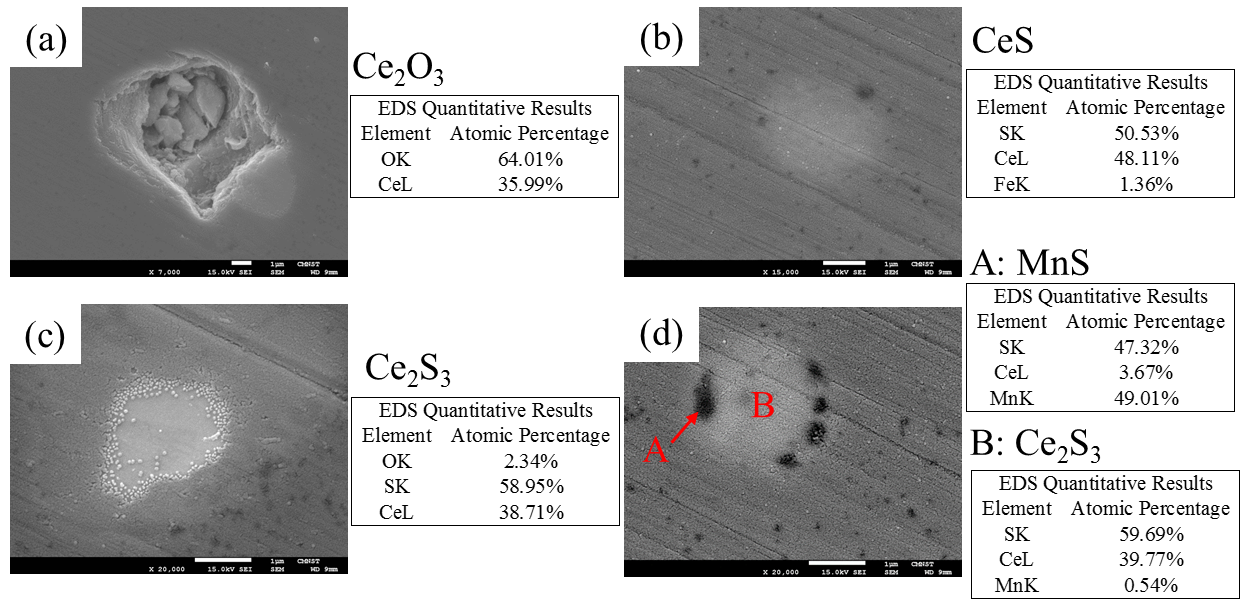


Supplementary Figure S5 Inclusion morphology for sample DM-3


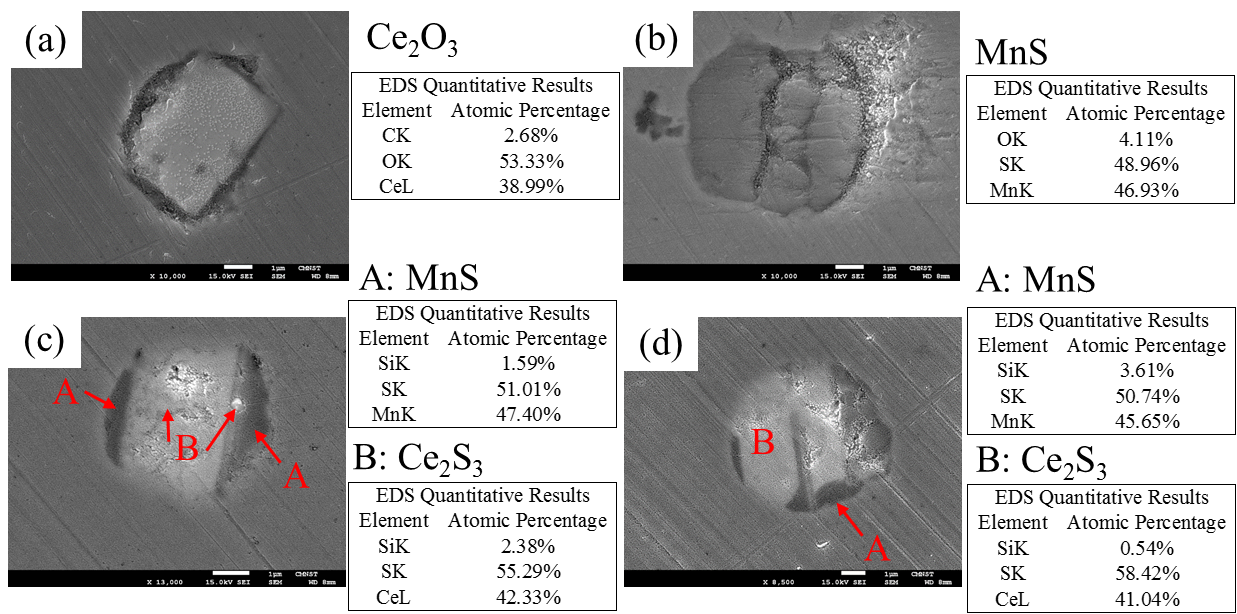


Supplementary Figure S6 Inclusion morphology for sample DM-4


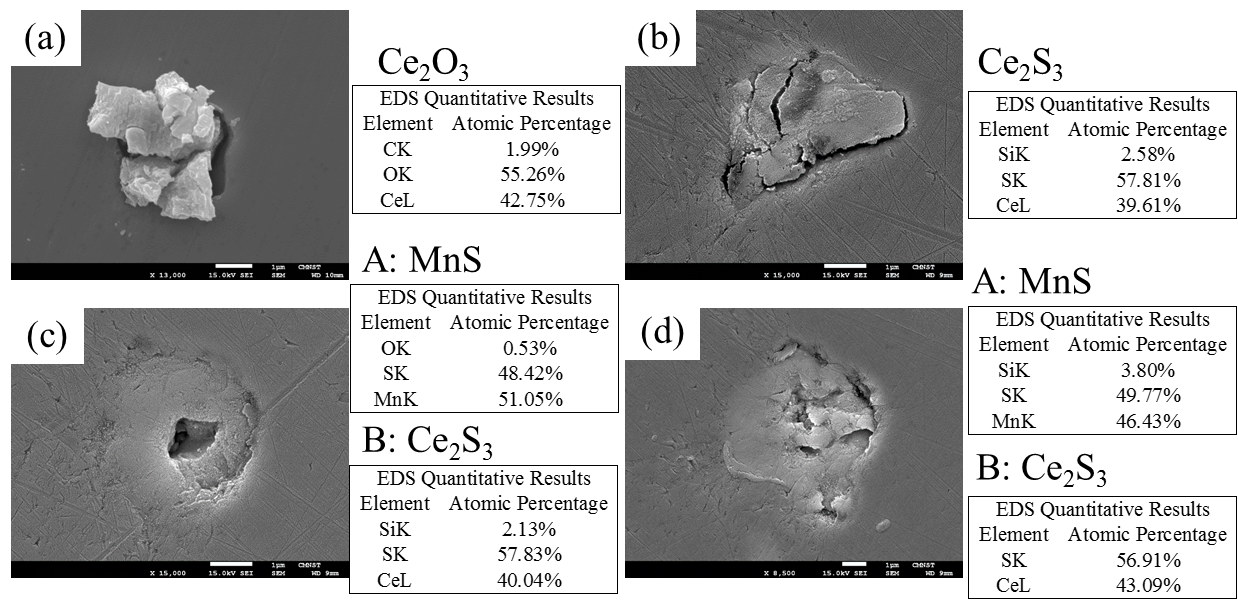


Supplementary Figure S7 Inclusion morphology for sample DM-5


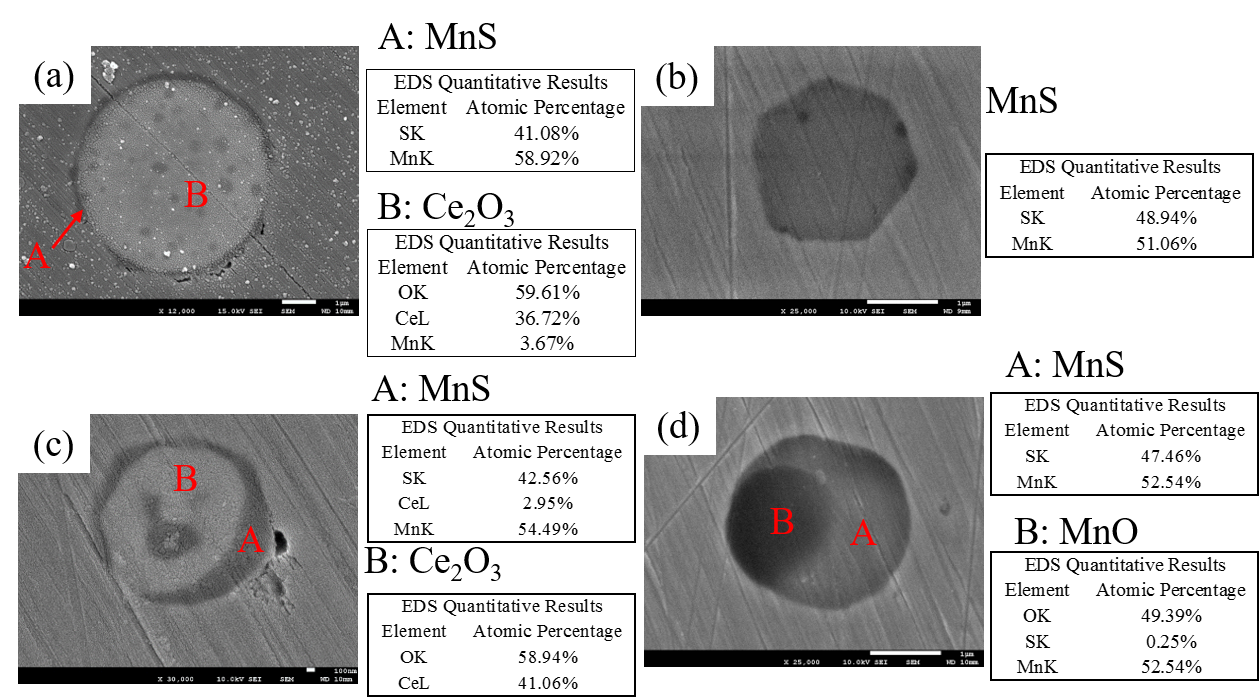


Supplementary Figure S8 Inclusion morphology for sample DM-6

*
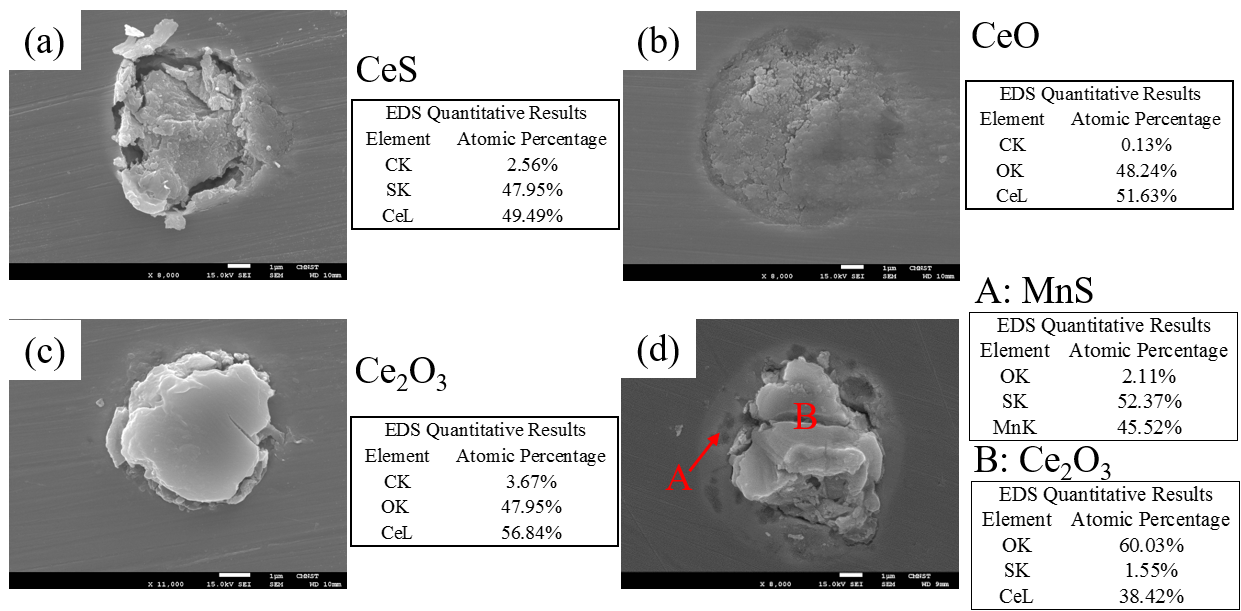
*

Supplementary Figure S9 Inclusion morphology for sample DM-7


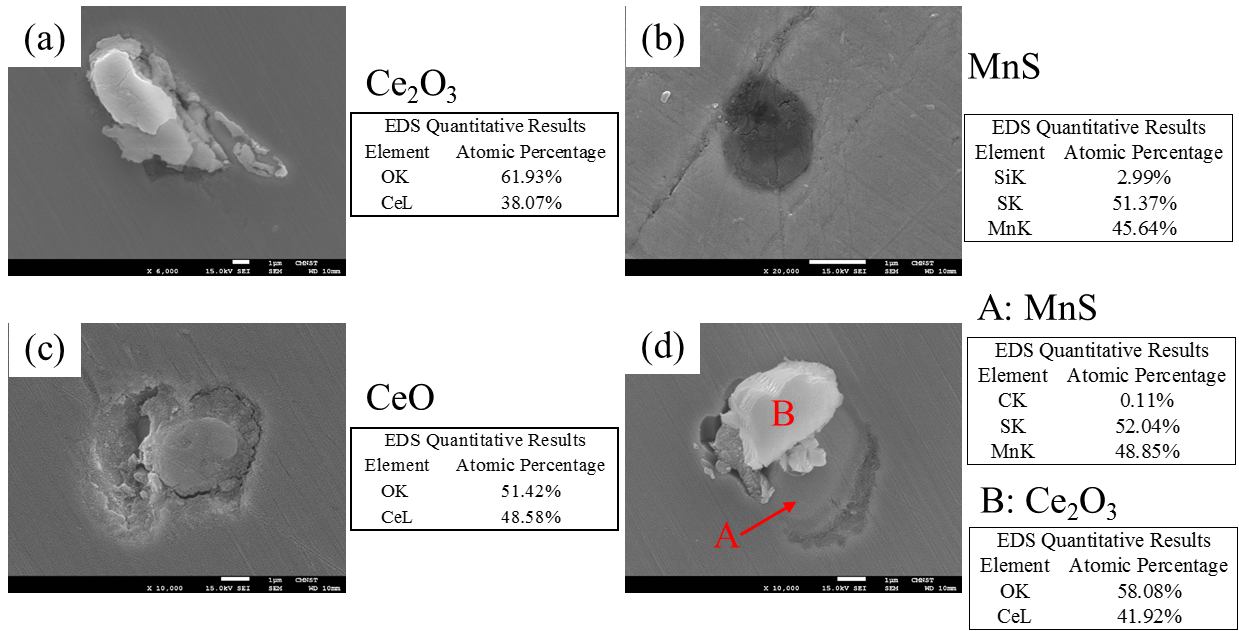


Supplementary Figure S10 Inclusion morphology for sample DM-8


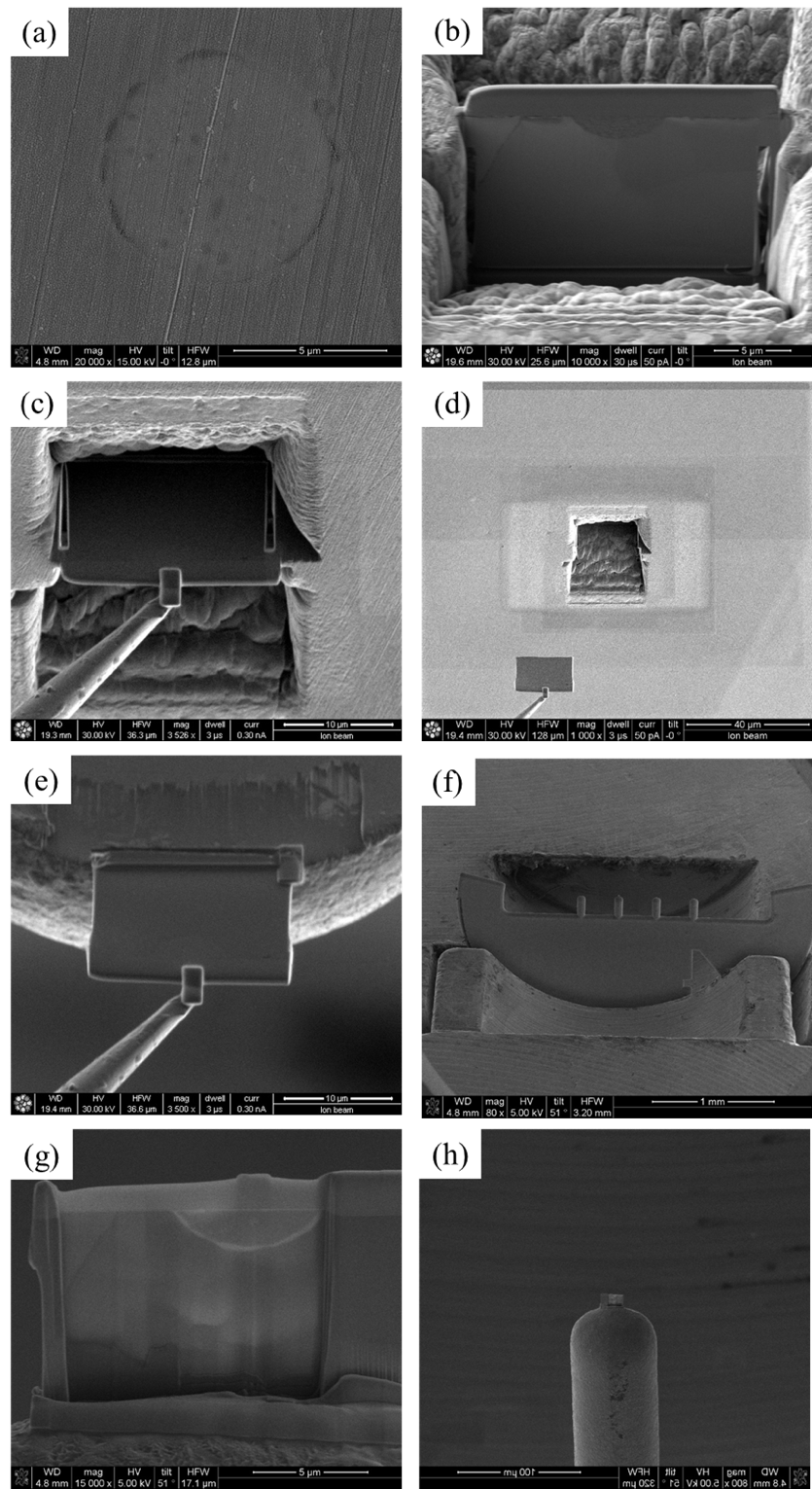


Supplementary Figure S11 Schedule of TEM sample preparation for the inclusion covered by MnS from sample DM-6
